# Semi-Parametric Spatial Joint Modeling of HIV and HSV-2 among Women in Kenya

**DOI:** 10.1371/journal.pone.0135212

**Published:** 2015-08-10

**Authors:** Elphas Okango, Henry Mwambi, Oscar Ngesa, Thomas Achia

**Affiliations:** 1 School of Mathematics, Statistics and Computer Science, University of KwaZulu-Natal, Private Bag X01, 3201 Pietermaritzburg, South Africa; 2 Division of Epidemiology and Biostatistics, School of Public Health, University of Witwatersrand, 27 St Andrews Road, 2193 Parktown, South Africa; 3 Mathematics and Informatics Department, Taita Taveta University College, P.O Box 635–80300, Voi, Kenya; University of Athens, Medical School, GREECE

## Abstract

Several diseases have common risk factors. The joint modeling of disease outcomes within a spatial statistical context may provide more insight on the interaction of diseases both at individual and at regional level. Spatial joint modeling allows for studying of the relationship between diseases and also between regions under study. One major approach for joint spatial modeling is the multivariate conditional autoregressive approach. In this approach, it is assumed that all the covariates in the study have linear effects on the multiple response variables. In this study, we relax this linearity assumption and allow some covariates to have nonlinear effects using the penalized regression splines. This model was used to jointly model the spatial variation of human immunodeficiency virus (HIV) and herpes simplex virus-type 2 (HSV-2) among women in Kenya. The model was applied to HIV and HSV-2 prevalence data among women aged 15–49 years in Kenya, derived from the 2007 Kenya AIDS indicator survey. A full Bayesian approach was used and the models were implemented in WinBUGS software. Both diseases showed significant spatial variation with highest disease burdens occurring around the Lake Victoria region. There was a nonlinear association between age of an individual and HIV and HSV-2 infection. The peak age for HIV was around 30 years while that of HSV-2 was about 40 years. A positive significant spatial correlation between HIV and HSV-2 was observed with a correlation of 0.6831(95% CI: 0.3859, 0.871).

## Introduction

According to the world health organization (WHO), more than 1 million people acquire sexually transmitted infections (STI) daily. WHO 2013 reports that more than 530 million (about 7.5%) have the virus that causes genital herpes or the herpes simplex virus type 2 (HSV-2) [1]. It was estimated that out of these, 123.7 million or 23% resided in sub-Saharan Africa, among whom 63% were women [2]. HSV-2 prevalence in the age group 15–49 in sub-Saharan Africa region ranges from 30% to 80% among women and from 10% to 50% among men [3]. People living with HIV were estimated to be about 35 million by the end of 2013 with 2.1 million new infections [4]. HSV-2 is associated with a two to threefold increased risk of HIV acquisition and an up to fivefold increased risk of HIV transmission per-sexual act, and may account for 40% to 60% of new HIV infections in populations where HSV-2 has a high prevalence [2], hence modeling these two diseases jointly may provide more insights on how these two diseases relate in Kenya. STIs can have serious consequences beyond the immediate impact of the infection itself, through mother-to-child transmission (MTCT) of infections and chronic diseases. Drug resistance is a major threat to reducing the impact of STIs worldwide [1].

Many studies have focused on monitoring HIV and HSV-2 trends in a country and comparison between countries using national averages [5]. These averages though important can hide the HIV and HSV-2 prevalence variability among administration units of a country and hence intervention strategies rolled out at national levels may not be effective at the administration level.

The national HIV and HSV-2 prevalence rate in Kenya within the adult population (15–64 years) was estimated to be as high as 5.6% and 7.1% respectively [6], with a wide gender and geographical variation. The HIV prevalence among women was 6.9% while among men was 4.4%. The North Eastern region had HIV prevalence of as low as 2.1% while regions around Lake Victoria and the Western had prevalence ranging from between 13%-25% [7]. The Kenya National AIDS and STI Control Program (NASCOP) in their Kenya AIDS Indicator Survey (KAIS) 2007 report stated that age had a non-linear relationship with HIV and HSV-2 prevalence, this is consistent with several studies which have shown that HIV and HSV-2 prevalence by age has a non-linear relationship assuming an inverted U shape [7,8]. HIV prevalence increases with age until it plateaus at between ages 25–35, then starts decreasing with increasing age. HSV-2 prevalence increases with age up to between ages 35–45 then begins to decline with increasing age.

Several studies have assumed that all the covariates in the study have a linear relationship with the response variable. This linear relationship may not hold for all variables as in our case age, which has a non-linear relationship with the response variable. Our objective is to perform a spatial joint modeling which allows for studying of the relationship between diseases and also between regions under study and at the same time capturing this nonlinear relationship. We extend the spatial semi parametric model based on penalized regression spline proposed by previous studies [9] to model HIV and HSV-2 jointly among women in Kenya.

## Methods

### Data

The data for this study was obtained from the Kenya AIDS Indicator Survey (KAIS) which was carried out by the Kenyan government with financial support from the United States President’s Emergency Plan for AIDS Relief (PEPFAR) and the United Nation (UN). The main aim of the survey was to obtain high quality data on the prevalence of HIV and Sexually Transmitted Infections (STI) among adults and to assess the knowledge of HIV and STIs in the population.

The sampling frame for KAIS was the National Sample Survey and Evaluation Program IV (NASSEP IV), it consisted of 1800 clusters comprising of 1260 rural and 540 urban clusters; of these, 294 rural and 141 urban clusters were sampled for KAIS. The overall design for KAIS 2007 was a stratified, two-stage cluster sampling design. The first stage involved selecting clusters from NASSEP IV, and the second stage involved the selection of household for KAIS with equal probability in the urban-rural strata within the districts. A sample of 415 clusters and 10,375 households were systematically selected for KAIS. A uniform sample of 25 households per cluster was selected using an equal probability systematic sampling method. The multilevel structure of the data in our analysis was accounted for through the random effects to account for within and between county variability.

The survey was twofold: A household questionnaire was used to collect the characteristics of the living environment and an individual questionnaire to collect information on demographic characteristics and the knowledge of HIV and STIs on men and women aged 15–64 years. A representative sample of households and individuals was selected from eight provinces in the country. Each individual was asked for consent to provide a venous blood sample for HIV and HSV-2 testing. More information on survey methodologies used in collecting the data is found in the final KAIS, 2007 report [7]. This study uses the 2007 data even though a new round of KAIS, 2012 [6] has been done. The final release of this new study had not been made hence the data was not available for use. This study uses the women’s data from the KAIS, 2007 survey. Information from 4864 women, aged 15–64 years who had provided venous blood for HIV and HSV-2 testing and also had full covariate information was used in the analysis. In the data, age was captured as both categorical and continuous while all other covariates were categorical. An initial exploratory data analysis was carried out using a univariate standard logistic regression model to determine the association of each single covariate with the outcome variable (HIV and HSV-2 status). These variables were categorized into four groups, namely: demographic, social, biological and behavioral.

From this initial analysis, education level, age at first sex, perceived risk, partners in the last one year, marital status, place of residence, STI status in the last one year and age of the respondent were found to be associated with HIV and HSV-2 infection. It was also established that age had a non-linear effect on HIV and HSV-2 infection, hence its continuous form (mean = 33.31, SD = 10.87) was used in the subsequent analyses.

### Ethical Statement

Ethical clearance was granted by the institutional review board of the Kenya Medical Research Institute (KEMRI) and the US Centers for Disease Control and Prevention. No ethical clearance was required from the University of Kwazulu-Natal or any other institution save for the aforementioned. The consent procedure, highlighted below, was approved by these two bodies.

Participants provided separate informed oral consent for interviews, blood draws and blood storage and, the interviewer signed the consent form to indicate whether or not consent was given for each part. An oral informed consent was given for participants in the age of 18–64 while for minors in the age group 15–17, an oral informed consent was obtained from a parent/guardian or other adult responsible for the youth’s health and welfare before the youth was asked for his/her consent. Only after the parent or guardian had agreed, was the consent of the adolescent sought.

Investigators in the study got a waiver of documentation of informed consent for all participants due to the fact that the research presented very minimal risk of harm to the individuals. The waiver did not adversely affect the rights and welfare of the participants, and the survey involved no procedures for which written consent is normally required outside the research context in Kenya.

### Statistical Model

A univariate standard logistic model was used to test the association of each single covariate with the outcome variable (HIV and HSV-2 status).The association was considered significant at 5% significance level. These results are shown in tables 1 and 2.

**Table 1:** Exploratory data analysis for HIV.

Variable	P-Value	Unadjusted OR
**Demographic characteristics**		
Place of residence (Ref Rural)		1
Urban	0.001	0.749(0.635, 0.884)
Age (Ref 15–19)	0.000	1
20–24	0.000	2.825(1.982, 4.026)
25–29	0.000	3.055(2.133, 4.375)
30–34	0.000	4.656(3.276, 6.618)
35–39	0.000	3.682(2.544, 5.328)
40–44	0.000	2.796(1.869, 4.181)
45–49	0.000	2.783(1.858, 4.169)
50–54	0.000	2.347(1.490, 3.696)
55–59	0.294	1.352(0.770, 2.375)
60–64	0.173	0.487(0.173, 1.371)
**Social Characteristics**		
Wealth Quantile (ref poorest)	0.525	1
Second	0.652	1.058(0.827, 1.353)
Middle	0.392	0.896(0.696, 1.153)
Fourth	0.564	1.074(0.843, 1.369)
Richest	0.592	0.938(0.741, 1.186)
Media access(Ref No)		1
Yes	0.257	0.913(0.781, 1.068)
Education level (Ref none)	0.000	1
Primary	0.386	1.078(0.910, 1.276)
Secondary	0.574	0.929(0.720, 1.200)
Higher	0.000	0.451(0.303,0 .671)
MaritalStatus(Ref Married, 1 partner)	0.000	1
Married, +2partners	0.001	1.536(1.192, 1.980)
Divorced/separated	0.000	2.503(1.960, 3.197)
Widowed	0.000	3.301(2.645, 4.120)
Never married	0.000	0.647(0.510,0 .820)
Perceived-Risk(Ref No risk)	0.000	1
Small Risk	0.000	0.325(0.231,0 .457)
Moderate Risk	0.000	0.447(0.335, 0.597)
Great Risk	0.574	0.916(0.676, 1.242)
Age-first-sex(Ref Never had sex)	0.000	1
Under 11	0.000	8.524(3.569, 20.358)
Between 12–14	0.000	10.162(5.774, 17.885)
Between 15–17	0.000	8.636(5.034, 14.817)
Over 18	0.000	4.870(2.833, 8.371)
**Biological characteristics**		
Had STI(Ref Yes)		1
No	0.000	0.406(0.277, 0.597)
Ever given birth(Ref Yes)		1
No	0.061	0.405(0.316,0 .519)
**Behavioral Characteristics**		
Partners in last 1 year (Ref No partner)	0.000	1
1 partner	0.034	1.021(0.314,0.812)
2 partners	0.665	1.232(0.771,3.433)
3 or more partners	0.999	2.455(1.759,11.233)
Travel away (didn’t stay away)	0.029	1
Stayed away 1–2 times	0.015	1.241(1.042, 1.477)
Stayed away 3–5 times	0.006	1.362(1.092, 1.698)
Stayed away 6–10 times	0.451	1.170(0.778, 1.761)
Stayed away > 11 times	0.748	0.894(0.451, 1.772)

**Table 2:** Exploratory data analysis for HSV-2.

Variable	P-Value	Unadjusted OR
**Demographic characteristics**		
Place of residence (Ref Rural)		1
Urban	0.000	0.823(0.746,0 .907)
Age (Ref 15–19)	0.000	1
20–24	0.000	2.745(2.254, 3.343)
25–29	0.000	4.374(3.591, 5.329)
30–34	0.000	6.794(5.559, 8.303)
35–39	0.000	8.299(6.739,10.220)
40–44	0.000	9.389(7.538, 11.694)
45–49	0.000	8.641(6.936, 10.765)
50–54	0.000	8.378(6.592, 10.649)
55–59	0.000	8.661(6.720, 11.162)
60–64	0.000	5.751(4.279, 7.729)
**Social Characteristics**		
Wealth Quantile (ref poorest)	0.051	1
Second	0.011	1.199(1.042, 1.381)
Middle	0.466	1.053(.916, 1.212)
Fourth	0.001	1.279(1.113, 1.469)
Richest	0.569	1.039(0.910, 1.186)
Media access(Ref No)		1
Yes	0.821	1.010(0.924, 1.104)
Education level (Ref none)	0.000	1
Primary	0.000	0.814(0.738, 0.898)
Secondary	0.000	0.704(0.610,0 .813)
Higher	0.000	0.457(0.381, 0.548)
Marital Status(Ref Married, 1 partner)	0.000	1
Married, +2partners	0.000	2.381(2.042, 2.778)
Divorced/separated	0.000	1.904(1.607, 2.256)
Widowed	0.000	3.238(2.719, 3.857)
Never married	0.000	0.292(0.257,0 .333)
Perceived-Risk(Ref No risk)	0.000	1
Small Risk	0.000	0.452(0.371,0 .551)
Moderate Risk	0.000	0.581(0.483, 0.699)
Great Risk	0.675	0.957(0.778, 1.177)
Age-first-sex(Ref Never had sex)	0.000	1
Under 11	0.000	12.572(7.554, 20.922)
Between 12–14	0.000	18.384(13.685, 24.697)
Between 15–17	0.000	15.053(11.477, 19.743)
Over 18	0.000	9.797(7.487, 12.818)
**Biological characteristics**		
Had STI(Ref Yes)		1
No	0.000	0.556(0.407,0 .760)
Ever given birth(Ref Yes)		1
No	0.052	0.187(0.163, 0.215)
**Behavioral Characteristics**		
Partners in last 1 year (Ref No partner)	0.009	1
1 partner	0.802	0.990(0.873,1.276)
2 partners	0.831	1.108(1.925, 6.294)
3 or more partners	0.938	0.535(0.699,1.434)
Travel away (didn’t stay away)	0.000	1
Stayed away 1–2 times	0.000	1.251(1.133, 1.380)
Stayed away 3–5 times	0.000	1.468(1.289, 1.672)
Stayed away 6–10 times	0.017	1.324(1.052, 1.665)
Stayed away > 11 times	0.198	1.258(0.887, 1.786)

Let *y*_*ijk*_ be the disease *k* status (0/1), *k* = 1 for HIV and *k* = 2 for HSV-2, for individual *j* in county *i*: *i* = 1,2,…, 46. In this notation *y*_*ij*1_ = 1 if individual *j* in county *i* is HIV positive and zero otherwise and *y*_*ij*2_ = 1 if individual *j* in county *i* is HSV-2 positive and zero otherwise. This study assumes the dependent variable *y*_*ijk*_ is bivariate Bernoulli distributed, i.e. *y*_*ijk*_|*p*_*ijk*_ ∼ *Bernoulli*(*p*_*ijk*_).

The vector ***X***_***ijk***_ = (*x*_*ij*1_, *x*_*ij*2_,…, *x*_*ijp*_)' contains *p* continuous independent variables and ***W***_***ijk***_ = (*w*_*ij*1_, *w*_*ij*2_,…, *w*_*ijr*_)' contains *r* categorical independent variables with the first component accounting for intercept. In this study, *p* = 1(*age*) and *r* = 8.

The unknown *E*(*y*_*ijk*_) = *p*_*ijk*_ relates to the independent variable as follows:

$$\begin{array}{l} h(p_{ij1})=X^{T}\beta_{1}+W^{T}\gamma_{1}\;,\;\text{for HIV and}\\ h(p_{ij2})=X^{T}\beta_{2}+W^{T}\gamma_{2}\;\text{for HSV}-2. \end{array}$$

Where *h*(.) is a logit link function, **β** is a *p* dimensional vector of regression coefficients for the continuous independent variables, and **γ** is a *r* dimensional vector of regression coefficients for the categorical independent variables. An extension to a semi parametric model utilizing the penalized regression spline approach and convolution model was employed in order to cater for both the non-linear effects of the continuous covariates and the spatial autocorrelation in the data.

The penalized regression spline approach relaxed the highly restrictive linear predictor by a more flexible semi-parametric predictor, defined as:

$$\begin{array}{l} h\left(p_{ij1}\right)=\sum_{t=1}^{p}f_{t}\left(x_{ijt}\right)+f_{spat}\left(s_{i1}\right)+W^{T}\gamma_{1}\;\text{for HIV and}\\ h\left(p_{ij2}\right)=\sum_{t=1}^{p}f_{t}\left(x_{ijt}\right)+f_{spat}\left(s_{i2}\right)+W^{T}\gamma_{2}\;\text{for HSV}-2 \end{array}$$

The function *f*_*t*_(.) is a non-linear twice differentiable smooth function for the continuous covariate and *f*_*spat*_(*s*_*i*_) is a factor that caters for the spatial effects of each county. This study utilized the convolution which assumes that the spatial effect can be decomposed into two components: spatially structured and spatially unstructured components i.e. *f*_*spat*_(*s*_*ik*_) = *f*_*str*_(*s*_*ik*_) + *f*_*unstr*_(*s*_*ik*_), *k* = 1,2 [9,10]. The spatially unstructured random effects cover the unobserved covariates that are inherent within the counties or the correlation within the counties e.g. common cultural practices, climate, cultures etc. while the spatially structured random effect accounts for any unobserved covariates which vary spatially across the counties, this is called spatial autocorrelation and it is technically defined as the dependence due to geographical proximity. The final model is expressed as:

$$\begin{array}{l} h\left(p_{ijk}\right)=\sum_{t=1}^{p}f_{t}\left(x_{ijt}\right)+f_{str}\left(s_{ik}\right)+f_{unstr}\left(s_{ik}\right)+W^{T}\gamma_{k},\\ with\;k=1\;for\;HIV\;and\;k=2\;for\;HSV-2 \end{array}$$

### Parameter Estimation

This study used a full Bayesian approach in estimation and parameters were assigned appropriate prior distributions as will be discussed in the priors section.

### The Penalized regression spline

Several studies have discussed extensively the methods for estimating the smooth function *f*_*t*_(.) [11,12]. In this study we utilize the penalized regression splines proposed by Eliers and Marx [13]. Here, the assumption is that the effect of the continuous covariates can be approximated using the polynomial spline. They assumed that the smooth function *f*_*t*_(.) can be estimated by a spline of degree *l* with *K* equally spaced knots, *x*_*p*,min_ = *ψ*_*p*1_ < *ψ*_*p*2_ ⋯ *ψ*_*pk*−1_ < *ψ*_*pK*_ = *x*_*p*,max_ giving:

$$f(x,\theta )=\phi_{0}+\phi_{1}x+\cdots \phi_{p}x^{l}+\sum_{k=1}^{K}b_{k}{\left(x-\psi_{k}\right)}_{+}^{l}\;,$$

where, **θ** = (*ϕ*_0_, *ϕ*_1_,⋯*ϕ*_*p*_, *b*_1_, *b*_2_,⋯*b*_*K*_)′ and (Λ−Ω)_+_ is equal to (Λ−Ω) if (Λ−Ω) is positive and zero otherwise.

This study uses a quadratic spline (*l* = 2) with 20 knots to ensure flexibility and takes the *k* ^*th*^ knot to be defined as the sample quantile of the continuous independent variable obtained by the probability equal to $\frac{k}{K+1}$. Green and Silverman [14] suggested a roughness penalty $-\frac{1}{2}\lambda \int_{x\;\text{min}}^{x\;\text{max}}{\left[f\prime \prime (x)\right]}^{2}dx$ imposed in the log-likelihood to avoid getting a smooth function which “wiggles” to much, yielding the penalized log-likelihood function given by:

$$L=l(y,\theta ,\gamma )-\frac{1}{2}\lambda \int_{x\;\text{min}}^{x\;\text{max}}{\left[f\prime \prime (x)\right]}^{2}dx,\;\text{where}\;\lambda \;\text{dictates the balance between flexibility and smoothness}.$$

### Prior distributions

The nearest neighbor multivariate Gaussian Markov random field (GMRF) is used as a prior distribution for the spatially structured effects ***f***_***str***_(***s***_***i***_) = (*f*_*str*_(*s*_*i*1_),*f*_*str*_(*s*_*i*2_))^*T*^. This is specified as:

$$f_{str}\left(s_{i},s_{j}\right)∼MCAR\left(1,\sum \right)\;\text{where},\sum \;\text{is the covariance matrix inducing correlation}.$$

Spatially, two regions are defined as neighbors if they share a border, otherwise they are not. Besag et al and Mardia et al discussed the univariate form of MCAR and conditions under which the conditional multivariate distributions uniquely determine the corresponding multivariate joint probability density function respectively [15,16]. Using these results, Carlin and Banerjee developed the MCAR [17]. Readers are also directed to the works of Gelfand and Vounatsou on the MCAR [18]. The unstructured spatial effects were assumed to follow a Multivariate Gaussian prior i.e. $f_{unstr}(s_{i},s_{j})|\tau_{unstr}^{2}∼MVN(0,\tau_{unstr}^{2})$. Inverse gamma distributions were assigned to the variance hyper parameters as:

$$\tau_{str}^{2}∼IG(0.0001,0.0001)\;\text{and}\;\tau_{unstr}^{2}∼IG(0.0001,0.0001).$$

The fixed effects coefficients were given the following prior distributions:

$$\begin{array}{l} \varphi_{0},\varphi_{1},\cdots \varphi_{p}∼N(0,10^{6}),\gamma_{1},\lambda_{2},\cdots \lambda_{r}∼N(0,10^{6}),b_{k}∼N(0,\tau_{b}^{2})\;\text{and}\\ \tau_{b}^{2}\sim IG(0.0001,0.0001),\beta_{1},\beta_{2}∼N\left(0.01,0.01\right)\;\text{being the intercepts}. \end{array}$$

### Posterior Distribution

The posterior distribution is obtained by updating the prior distribution with the observed data and hence it is the distribution of the parameters after observing the data. This posterior distribution is what gives samples for Bayesian inference. Markov chain Monte Carlo (McMC) overcomes the problem of high dimensionality as it allows for direct sampling from this posterior distribution repeatedly and estimates such as mean and median are calculated from these simple data summaries.

Assuming Conditional independence between the response variable and the hyper parameters, the posterior distribution for the Bernoulli model is given by:

$$\begin{array}{l} P_{post}(\phi ,\lambda ,b,\tau^{2}|y)\alpha \;L(y|\phi ,\lambda ,b,\tau^{2})P_{pri}(\phi ,\lambda ,b,\tau^{2})\\ =\prod_{i}\prod_{j}\;L(y_{ij}|\theta ,\lambda ,\tau^{2})\prod_{k=1}^{p}\left[P(b_{k}|\tau_{k}^{2})P(\tau_{k}^{2})\right]\times \\ \;\prod_{j=1}^{r}\left[P(\gamma_{j}|\tau_{j}^{2})P(\tau_{j}^{2})\right]\times \\ \;P(f_{str}|\tau_{str}^{2})P(\tau_{str}^{2})P(f_{unstr}|\tau_{unstr}^{2})P(\tau_{unstr}^{2}) \end{array}$$

All the analyses in this study were carried out using WinBUGS 14 [19]. In the implementation, 20,000 Markov chain Monte Carlo (McMC) iterations for each model was run, with the initial 10,000 discarded to carter for the burnin period. The 10,000 iterations left were used for assessing the convergence of the McMC and parameter estimation.

### Model Diagnostics

The models were compared using the deviance information criterion (DIC) suggested by Spiegelhalter et al [20]. The best fitting model is one with the smallest DIC. The DIC value is obtained as: $DIC=\bar{D}(\theta )+pD$, where $\bar{D}$ is the posterior mean of the deviance that measures the goodness of fit while *pD* gives the effective number of parameters in the model which penalizes for complexity of the model. In DIC, low values of $\bar{D}$ indicate a better fit while small values of *pD* indicate model parsimony. One challenge with the DIC is, how big the difference in DIC values of two competing models needs to be in order to declare one model as being better than the other is not well defined. Studies have shown that a difference of 3 in DIC between two models cannot be distinguished while a difference of between 3 and 7 can be weakly differentiated [20,21].

## Data Analysis

This study investigated four sets of models in order to get an insight on the effect of the covariates, the unobserved effects on the distribution and relationship between HIV and HSV-2 in Kenya based on the female data. Studies have discussed these classes of models and their advantages over classical models [22–24]

$$\begin{array}{l} Model_{1}\;:\;logit(\rho_{ij1})=\beta_{01}+f(age)+w^{T}\gamma \;for\;HIV\\ \;logit(\rho_{ij2})=\beta_{02}+f(age)+w^{T}\gamma \;for\;HSV-2\\ Model_{2}\;:\;logit(\rho_{ij1})=\beta_{01}+f(age)+w^{T}\gamma +f_{unstr}\left(s_{i1}\right)\;for\;HIV\\ \;logit(\rho_{ij2})=\beta_{02}+f(age)+w^{T}\gamma +f_{unstr}\left(s_{i2}\right)\;for\;HSV-2\\ Model_{3}\;:\;logit(\rho_{ij1})=\beta_{01}+f(age)+w^{T}\gamma +f_{str}\left(s_{i1}\right)\;for\;HIV\\ \;logit(\rho_{ij2})=\beta_{02}+f(age)+w^{T}\gamma +f_{str}\left(s_{i2}\right)\;for\;HSV-2\\ Model_{4}\;:\;logit(\rho_{ij1})=\beta_{01}+f(age)+{w\prime }_{\mathrm{ij}}\gamma +f_{unstr}\left(s_{i1}\right)+f_{str}\left(s_{i1}\right)\;for\;HIV\\ \;logit(\rho_{ij2})=\beta_{02}+f(age)+{w\prime }_{\mathrm{ij}}\gamma +f_{unstr}\left(s_{i2}\right)+f_{str}\left(s_{i2}\right)\;for\;HSV-2 \end{array}$$

### Model 1

This is a model of fixed categorical covariates which are assumed to have linear effects on the response variable namely, education level, age at first sex, perceived risk, partners in the last one year, marital status, place of residence, STI status in the past one year, number of times one had stayed away from home in the past one year and one continuous covariate, age, modeled with a non-linear smooth function. Results from [5,25] supports modeling age with a non-linear smoothing prior. Model 1 does not take into account the spatially structured and the spatially unstructured random effects and the two diseases are modeled independently.

### Model 2

This is an additive model that assumes linear effects of the categorical covariates listed in model 1 above, non-linear effect of the continuous covariate age and spatially unstructured random effect which cover the unobserved covariates that are inherent within the counties. Here the joint modeling is initiated by the multivariate normal distribution.

### Model 3

This model explores the effect of the linear covariates listed in model 1 above, non-linear covariate age and spatially structured random effect which accounts for any unobserved covariates which vary spatially among counties. The joint modeling is initiated by the multivariate conditional autoregressive model.

### Model 4

Examines the effects of the nonlinear effects of age, linear effects of the categorical covariates and a convolution of spatially structured and spatially unstructured random effect, and the joint modeling is initiated by both the multivariate normal distribution and the multivariate conditional autoregressive model.

## Results

### Model assessment and comparison

Table 3 gives the nesting nature of the models under study. Model 1 basically examines the linear and nonlinear effects of the covariates, model 2 extends model 1 to include spatially unstructured random effects, model 3 extends model 1 to include spatially structured random effects and finally model 4 is model 1 plus both structured and unstructured random effects.

**Table 3:** Nesting nature of the models under study.

Model	Nonlinear effect of age	Linear effects of categorical covariates	Spatially unstructured random effects	Spatially structured random effects
***M***_**1**_	✓	✓	_	_
***M***_**2**_	✓	✓	✓	_
***M***_**3**_	✓	✓	_	✓
***M***_**4**_	✓	✓	✓	✓

Table 4 presents model diagnostics for the four fitted models. The model with the smallest DIC provides a better fit. However studies have reported that a difference of 3 in DIC between two models cannot be distinguished while a difference of between 3 and 7 can be weakly differentiated [20,21]. This implies therefore that model 2 and model 4 are indistinguishable since the difference in their DIC is less than 3. We therefore present and discuss results based on model 4 as it captures both spatially structured and unstructured random effects.

**Table 4:** Models comparison.

	Model1		Model2		Model3		Model4	
	HIV	HSV −2	HIV	HSV −2	HIV	HSV −2	HIV	HSV −2
**Individual *pD***	23.425	25.424	32.755	56.869	43.211	57.869	43.149	58.133
**Individual** $\bar{D}\left(\theta \right)$	2447.41	6040.86	2319.64	5732.09	2312.85	5733.05	2308.84	5733.01
**Individual DIC**	2470.83	6066.29	2252.40	5788.96	2356.06	5790.91	2351.99	5791.14
**Total DIC**	8537.27		8141.36		8146.97		8143.13	

### Fixed effects

Table 5 gives the posterior estimates of the odds ratios (OR) and their corresponding 95% credible intervals (CI) for the categorical covariates which were assumed to have linear effects on HIV and HSV-2 based on model 4.

**Table 5:** Parameter estimates of based on Model 4.

Covariates	HIV	HSV-2
**Demographic Characteristics**		
Place of residence (ref rural)	1	1
Urban	1.592 (1.116, 2.211)	1.904 (1.549, 2.313)
**Social characteristics**		
Marital status(ref Married,1 partner)	1	1
Married, 2 partners	0.9232 (0.6231, 1.32)	1.934 (1.532, 2.427)
Divorced/separated	2.78 (1.81, 4.091)	2.504 (1.818,3.365)
Widowed	4.603 (2.598, 7.477)	3.11 (1.856, 5)
Never Married	1.376 (0.8911, 2.016)	0.9912 (0.7627, 1.275)
Perceived risk(ref No risk)	1	1
Small Risk	0.4926 (0.3148, 0.7216)	0.6647 (0.5111, 0.8345)
Moderate risk	0.5361 (0.3625,0.7541)	0.7051 (0.5486, 0.8699)
Great risk	0.8726 (0.5901, 1.239)	0.9545 (0.7299, 1.201)
Age at first sex(ref Over 18)	1	1
Under 11	2.702 (0.8462, 6.095)	2.196 (0.9663, 4.342)
Between 12–14	1.691 (1.153 2.393)	2.055 (1.604, 2.575)
between 15–17	1.407 (1.063, 1.851)	1.61 (1.373, 1.866)
Stay away(ref > 11 times)	1	1
Didn't stay away	1.282 (0.5137, 2.594)	1.22 (0.7116,2.046)
1–2 times	1.179 (0.474, 2.351)	1.29 (0.754, 2.194)
3–5 times	1.725 (0.6809, 3.469)	1.437 (0.8379, 2.472)
6–10 times	1.368 (0.4605, 3.039)	1.232 (0.6684, 2.176)
Education(ref Higher)	1	1
None	2.425 (1.425, 4.199)	2.184 (1.662, 2.851)
Primary	2.168 (1.26, 3.715)	2.072 (1.581, 2.666)
Secondary	2.343 (1.274, 4.086)	1.808 (1.346, 2.383)
**Behavioral Characteristics**		
Partners in last 1 year(3 or more)	1	1
1 partner	1.283 (0.235, 5.762)	1.896 (0.4114, 6.478)
2 Partners	1.992 (0.3234, 8.993)	2.528 (0.5068, 8.682)
**Biological Characteristics**		
STI(ref no)	1	1
Yes	1.57 (0.8439, 2.611)	1.382 (0.9156, 1.995)
Random effects		
Spatially unstructured (*τ*_*unstr*_)	0.143(0.000,0.645)	0.167(0.012,0.533)
Spatially structured (*τ*_*str*_)	0.141(0.024,0.982)	0.159(0.412,1.323)
Spline Coefficients *τ*_*b*_	5674(1003,7554)	7683(870.8,9356)
Correlation (HIV-HSV-2)	0.6831(0.3859,0.871)	

Place of residence, marital status, education level, perceived risk, age at first sex, number of partners in the last year, if an individual had STI in the last 12 months and the number of times an individual had stayed away from home in the last one year were found to be significantly associated with HIV and HSV-2 infection.

### HIV

Place of residence (urban/rural) was found to be associated with HIV infection among women. The odds of HIV infection among women staying in urban areas was 1.592 times as likely as that of women living in rural areas (OR: 1.592, 95% CI: 1.116 to 2.211). Marital status was also significantly associated with HIV infection. The odds of HIV infection among divorced/separated women was 1.78 times higher than women who were married with one partner (OR: 2.78, 95% CI: 1.81 to 4.091). Women who had never been married were found to be 1.376 as likely to be HIV positive as women who were married with one partner

(OR: 1.376, 95% CI: 0.8911 to 2.016), though it is not significant. Widowed women were 3.603 times more likely to be HIV positive than women who were married to one partner (OR: 4.603, 95% CI: 2.598 to 7.477). Those women who had some perceived risk of HIV infection (small risk, Moderate risk, Great risk) were less likely to be HIV positive than those who had no perceived risk. Age at first sex is negatively associated with HIV infection. The likelihood for HIV was higher for those women who had had their first sex before age 11 as compared to those who had had their first sex after age 18, and this was not significant as indicated by the odds ratio and its corresponding credible interval (OR: 2.702, 95% CI: 0.8462 to 6.095). The chance of testing positive for HIV was 0.691 times higher for women who had had their first sex between ages 12–14 years than those who had their first intercourse after age 18 (OR: 1.691, 95% CI: 1.153 to 2.393). Education level was also found to be associated with HIV infection. Those women with no education were 1.425 more likely to test positive for HIV than those with higher education (OR: 2.425, 95% CI: 1.425 to 4.199). The chance of HIV infection was lowest among women with higher education. Individuals who contracted an STI in the last 12 months were found to be 1.57 times as likely to test positive for HIV as those who had not (OR: 1.57, 95% CI: 0.8439 to 2.611).

### HSV-2

Place of residence (urban/Rural) was found to be associated with HSV-2 infection. Women who resided in urban locations were 1.904 times as likely to test HSV-2 positive as those residing in rural areas (OR: 1.904, 95% CI: 1.549 to 2.313). Marital status was also found to be associated with HSV-2 infection among women. The odds of testing positive for HSV-2 was 0.9912 times as less likely for those women who were never married as for those who were married with one partner (OR: 0.9912, 95% CI: 0.7627 to 1.275). Women who were married with more than one partner were 1.934 times as likely to test positive for HSV-2 as those who were married with one partner (OR: 1.934, 95% CI: 1.532 to 2.427). Divorced/separated women were 1.504 times more likely to test positive for HSV-2 than those women who were married with one partner (OR: 2.504, 95% CI: 1.818 to 3.365). Widowed women were most likely to test positive for HSV-2. Widowed women were 3.11 as likely to test positive for HSV-2 as those women who were married with one partner (OR: 3.11, 95% CI: 1.856 to 5.000). HSV-2 infection is positively associated perceived risk. The chance of testing positive increased with increasing perceived risk. However women who had some perceived risk were less likely to test positive for HSV-2 as compared to those who felt they had no risk. Women who perceived great risk of infection were 0.9545 as less likely to test positive for HSV-2 as those who felt no risk at all, although this was not significant (OR: 0.9545, 95% CI: 0.7299 to 1.201). The likelihood of infection on women that had a perception of moderate risk was 0.7051 as less likely as for those women who felt not at risk (OR: 0.7051, 95% CI: 0.5486 to 0.8699). Women who had their first intercourse below age 11 years were 1.196 times more likely to test positive for HSV-2 than those who had their first intercourse after age 18. The odds of women who had had their first sexual intercourse between ages 12 and 14 to be infected with HSV-2 were 2.055 times as higher as those who had engaged in their first intercourse after age 18.

HSV-2 infection is negatively related with education. The likelihood of HSV-2 infection was 1.184 higher for those with no education as compared with those who had attained higher education, (OR: 2.184, 95% CI: 1.662 to 2.851). Women who had primary education were 1.072 times more likely to test positive for HSV-2 than those with higher education (OR: 2.072, 95% CI: 1.581 to 2.666).

Another finding of this study is that those women with higher education qualification were less likely to test positive for both HIV and HSV-2.

### Nonlinear effects of age

**Fig 1:**
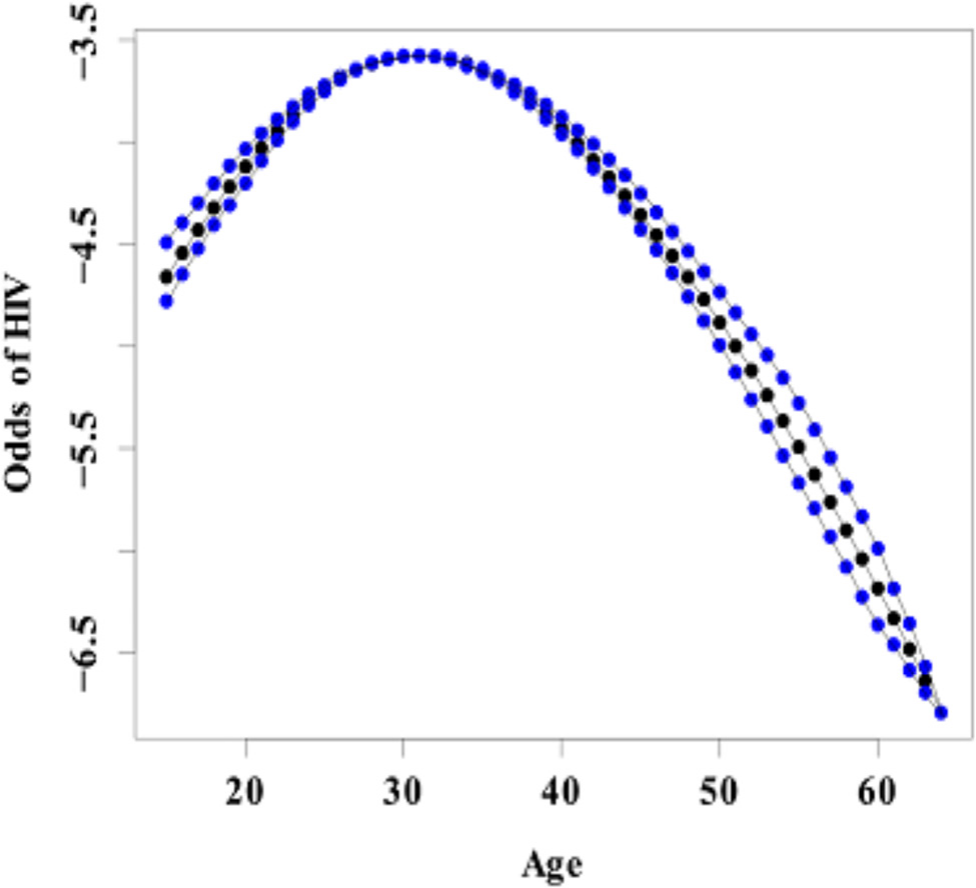
Estimated mean of the Nonlinear effect of age (in black) on HIV infection and the corresponding 95% credible interval(blue).

**Fig 2:**
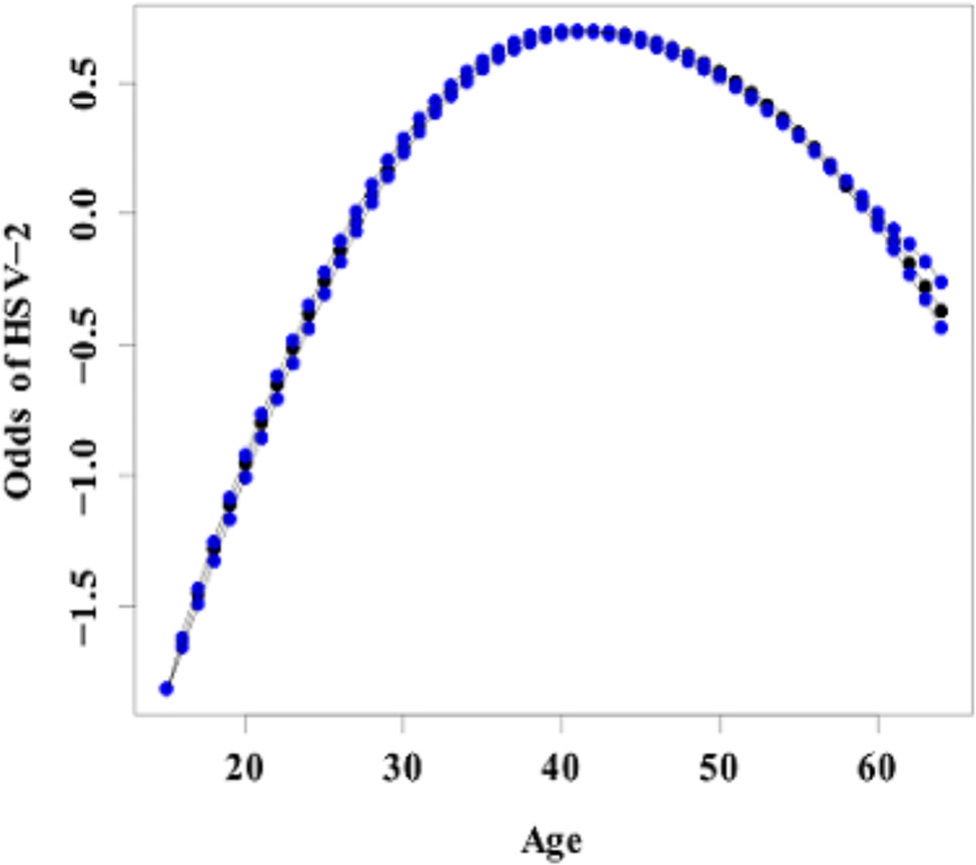
Estimated mean of the Nonlinear effect of age (in black) on HSV-2 infection and the corresponding 95% credible interval (in blue).

Figs 1 and 2 show the nonlinear association between age of an individual and HIV infection and age of an individual and HSV-2 infection. The figures give the posterior mean of the smooth function and their corresponding 95% CI. From the figures it is evident that there is a nonlinear relationship between age and HIV and HSV-2 infection. An assumption of linear relationship would have led to miss leading results and subsequently wrong interpretations. The chance of HIV infection increases with age up to an optimum age of about 30 years then starts declining with increase in age. For HSV-2, the likelihood of infection increases with age up to an optimum age of about 40 years then starts to decline thereafter with increasing age. The result depict that the prevalence of HIV picks earlier in age than HSV-2. Early age at first sex often times leads to individuals developing risky sexual behaviors like having multiple partners and not using protection as the individual grows older increasing the chances of getting HIV or HSV-2 with increasing age. HIV and HSV-2 prevalence also increases with age from between age 15 and 30 as this is the time the youth is in risky behavior such as unprotected sex and having multiple partners. HIV and HSV-2 prevalence stagnates at 30 and 40 respectively before dropping and this could be assumed to be the age where women have either settled in marriage and are practicing safe sexual relationships or are becoming less active sexually hence the declining prevalence. The late peaking of HSV-2 could be attributed to its late detection as they have mild to no symptoms at all or their symptoms may be mistaken for other conditions. This carries with it a negative public health implication in that, this is the age when the youth is most active and more willing to take risks. High prevalence in this age group implies high number of new infections and hence curbing HIV and HSV-2 becomes more difficult. Strategies to delaying age at first sex, practicing responsible sexual behavior will help reduce the prevalence of these two diseases.

### Joint Spatial effects

**Fig 3:**
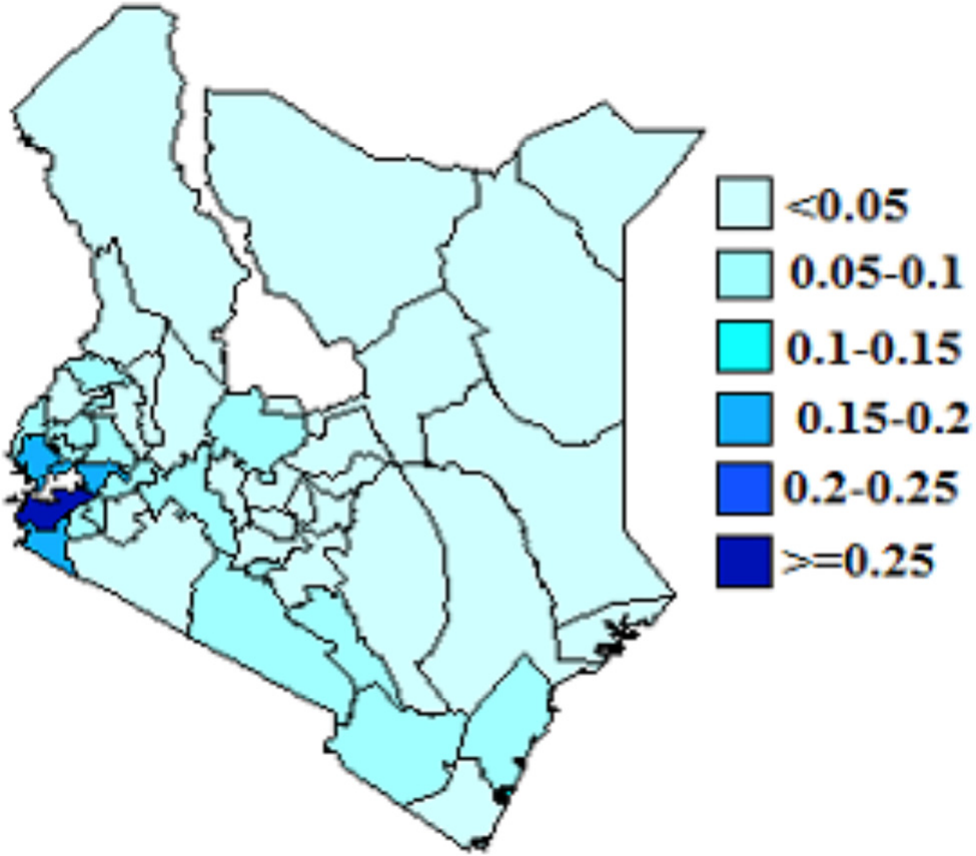
Residual spatial effect of county on HIV.

**Fig 4:**
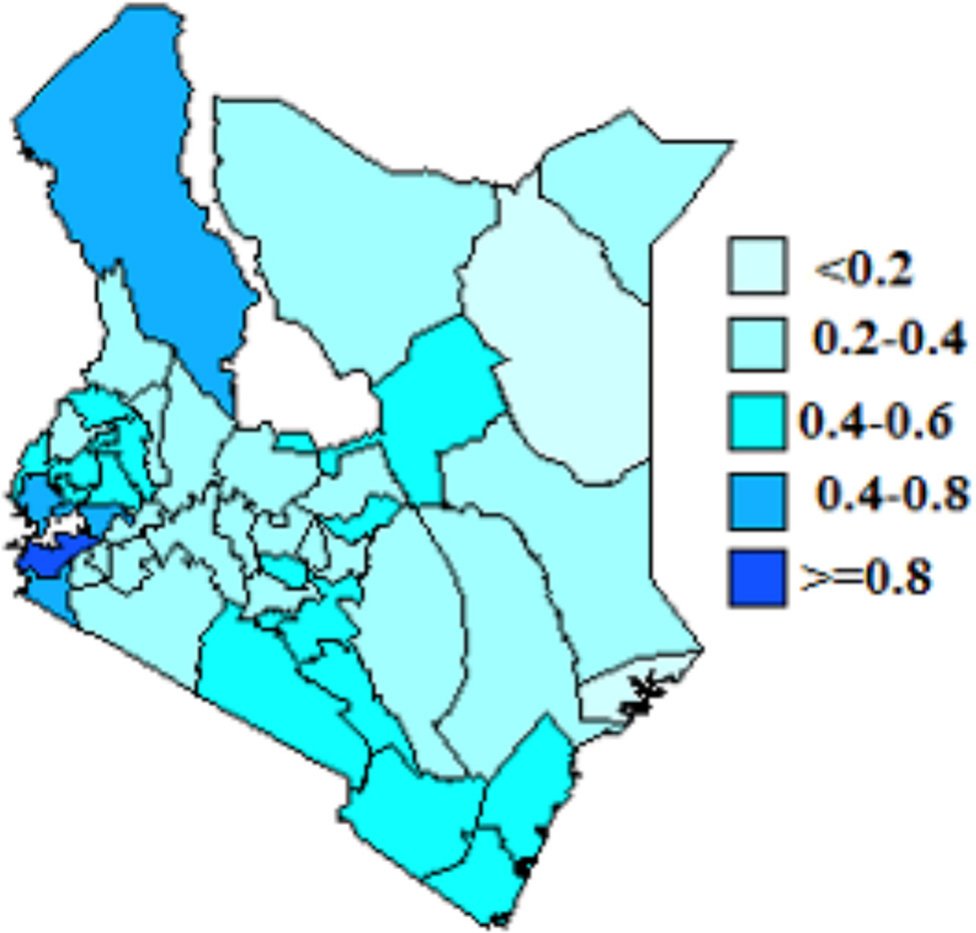
Residual spatial effect of county on HSV-2.

We present spatial effects based on model 4. These are shown in Figs 3 and 4. From the figures, counties with dark blue shading show high association of HIV and HSV-2 infection while light blue shading indicate low association of HIV and HSV-2 infection. The figures show spatial variation of HIV and HSV-2. From Fig 3, counties in the Western and around Lake Victoria regions had high HIV prevelance. Counties in the North Eastern region had low HIV prevelance. Siaya, Homabay,Migori and Kisumu counties recorded the highest HIV prevelance. In Fig 4, Siaya, Homabay, Migori, Kisumu and Turkana counties recorded the highest HSV-2 prevelance. HSV-2 prevelance was higher than HIV prevelance and more spread than HIV.

## Discussion

This study utilizes a full Bayesian approach to perform a semi-parametric spatial joint modeling of HIV and HSV-2 in Kenya. In particular, we used these methods to analyze the regional variation, risk factors of HIV and HSV-2 and the association between HIV and HSV-2. The works of Eliers and Marx [21] on the B-splines, their construction and the penalized likelihood and that of Caroll and Rupert [22] on semi-parametric regression provide a basis for this study. In particular we model the non-linear effects using the penalized regression splines, in a semi parametric model exemplar, allowing for spatial variation in the response variables. The linearity assumption between the response variable and the covariates is limiting, unrealistic and can lead to misleading results in many situations. Semi parametric models are more flexible as they combine both parametric and semi parametric models hence enriching the standard parametric model by exploring the non-parametric domain while still keeping intact the linear structure [15].

Age was found to have a non-linear effect on both HIV and HSV-2. i.e. an inverted “U” shape. The likelihood of HIV infection among women increases with age up to about age 30 then reduces thereafter with increasing age. On the other hand the likelihood of HSV-2 infection increases with age up to about age 40 and then starts declining with age. These findings were consistent with other studies [25].

The spatial effects in the model are modeled using a Gaussian Markov Random Field (GMRF) while the spatially unstructured random effects are modeled using a zero mean Gaussian process [15,21]. Bayesian and non-Bayesian methods have been proposed for joint disease modeling [26,27]. The maximum likelihood (frequentist) approaches are not viable for these models due to the high complexity and intractability, hence the Bayesian inference, utilizing the McMC techniques is highly favored [9]. The computational limitations of the frequentist approach makes the Bayesian approach through the McMC algorithm more appealing as it is less cumbersome to implement. Bayesian approach allows for complex and flexible hierarchical modeling while providing more reliable estimates and predictions for many realistic epidemiological problems. While parameters are estimated similarly under the two methods, random effects variance estimates are generally attenuated under the frequentist approach compared to the Bayesian approach [10].

Place of residence was found to be significantly associated with HIV and HSV-2 infection among women when controlled for other covariates. Women in urban areas were more likely to be HIV and HSV-2 positive than women living in rural areas. Many studies have reported the effect of place of residence on HIV infection but with mixed conclusions [25,28]. From our study, HSV-2 infection was also more prevalent in Turkana County which is mostly rural when considering the prevalence at county levels. These findings could be used to inform area specific approaches and campaign strategies to help curb the prevalence of these two diseases.

Marital status was also significantly associated with HIV and HSV-2 infection. Women who had been married before and then divorced, separated or widowed were more likely to test positive for HIV and HSV-2 than those who were married with one partner or never been married. Widowed women were the most likely to test positive for HIV and HSV-2 in comparison to those who were married with one partner. This could be attributed to wife inheritance. Wife inheritance is a widespread cultural practice in sub-Saharan Africa that increases the risk of HIV acquisition and transmission [29,30]. The life expectancy of females is higher than that of males in most cases and countries, with the gap between sexes steading at 5 since 1990 [31], this in effect means that it is more likely that a man will die leaving behind his HIV/HSV-2 infected wife, and if she accepts to be inherited, she will pass it to her inheritor who will acquire the disease and pass it to the wife before dying and leaving them. These two widows will then be inherited by another individual/s and the chain goes on. In most cases these inheritors engage in concurrent sex and are polygamous with some having more than 2 wives.

This study also found that age at first sex was negatively associated with HIV and HSV-2 infection. Those who had had their first sexual contact before age 11 were more likely to test positive for HIV and HSV-2 than those who had had their first intercourse after age 18. Other studies have found similar results [8]. This knowledge can help in designing of prevention programs not only aiming at delaying the age at first sex but also addressing the factors leading to early sexual practices.

Women who had had STI in the last 12 months were also more likely to test positive for HIV and HSV-2. This has been documented in various studies [32–34]. Education level was found to be inversely related to HIV and HSV-2 infection. Those who had attained higher education qualification were less likely to test positive for HIV and HSV-2. This is consistent with other studies which reported similar results [33]. The introduction of free primary education and the subsequent subsidizing of secondary education is hoped to increase the number of people attaining higher education level [35].

HIV and HSV-2 infection were also found to be highly correlated, and this was significant: 0.6831(0.3859, 0.871). Counties with high HSV-2 prevalence had a high HIV prevalence too.

Spatial effects in the model account for unobserved variables that represent those variables that vary spatially. Identifying high prevalence areas and the relationship between HIV and HSV-2 can provide more insight that can be useful in coming up with tailor made campaigns and prevention strategies for specific regions. There was evidence of spatial variation of HIV and HSV-2 infection among counties. The highest prevalence rate for HIV was observed in Western part of the country and around Lake Victoria while highest prevalence for HSV-2 was observed in Western region, around Lake Victoria and Turkana. Availability of free software like R and WinBUGS makes the establishing and testing epidemiological hypothesis easier and the implementation of these complex models cheaper.

The major limitation for this study was that the data used for county estimation was collected when the country was still based on the old administrative units (provinces) however these new administrative units (counties) were formed by combining several districts together. This made it easy for the county where an individual belongs to be allocated easily since each district belongs to only one county. The knots used in the penalized spline regression were assumed to be fixed and were calculated as quantiles from the continuous variable age. A more flexible analysis can allow the knots to be data driven [36]. Another limitation for this study is that the data used for this study are from 2007 survey. A more recent KAIS survey has been conducted although it had not yet been made public by the time this study was carried out. The models introduced in this study can be replicated in other countries with similar data.

Future work can also allow for time trends to exploit subsequent surveys that collect data on the two infections.

## Supporting Information

S1 Text WinBUGS codes used in the analysis. (ODC)
